# Supplementary material for: Predicting survival of glioblastoma from automatic whole-brain and tumor segmentation of MR images
Source: Sci Rep. 2022 Nov 17;12:19744. doi: 10.1038/s41598-022-19223-3 (PMC9671967; doi:10.1038/s41598-022-19223-3)
Supplement: Supplementary file 1 — Supplementary Information. [file 41598_2022_19223_MOESM1_ESM.pdf]

Supplementary Information

1 Robustness to segmentation errors

To analyze the effect of segmentation errors on our results, we repeated some of our experiments on the main (Copenhagen) dataset, but this time after artificially increasing the Hd95 distances to simulate inaccuracies in automatically located segmentation boundaries. Specifically, we increased each computed Hd95 feature value by a random percentage drawn uniformly from the interval $[0, max_{\%}]$, for three levels of maximum error magnitude: $max_{\%} = 5\%$, 15%, and 25%. We repeated this procedure 100 times, each time training and testing our prediction models using Hd95 features tainted with a different error sample.

Table S1 and Fig. S1 show results for OS prediction obtained this way, using RSF models trained on the combination of tainted Hd95 features and clinical features. The middle column of Table S1 shows the obtained subject-level prediction performance (C-index), averaged over the 100 error samples and accompanied by the standard error of the mean. Compared to the corresponding result without the extra random noise (C-index of 0.669 (0.668−0.671), cf. Table 2 in the paper) the C-index we obtain is only about 0.01 lower for all three noise levels. We also repeated the risk group stratification experiment this way, averaging the survival prediction over the 100 error samples and dichotomizing the cohort as in the original experiment (cf. Fig. 6 in the paper). Fig. S1 shows the resulting Kaplan-Meier survival curves for each noise level, overlaid on those obtained in the original experiment in the paper (shown in dotted line) for comparison. The corresponding hazard ratios and P-values are shown in the right column of Table S1, and can be seen to not differ significantly from those in the original experiment (hazard ratio 2.65 (1.85 - 3.79), $P = 10^{-8}$).

From these results, we conclude that the proposed methods are fairly robust to errors in the automatic segmentation procedure.

$max_{\%}$	C-index	Hazard Ratio
5%	0.657 (0.656-0.658)	2.54 (1.78 - 3.63) $P = 10^{-7}$
15%	0.657 (0.656-0.658)	2.65 (1.85 - 3.79) $P = 10^{-8}$
25%	0.658 (0.657-0.659)	2.58 (1.81 - 3.69) $P = 10^{-7}$

Table S1: Results for subject-level OS prediction performance and risk group stratification on the Copenhagen dataset when segmentation errors are artificially introduced. The features included were the clinical and Hd95 features.

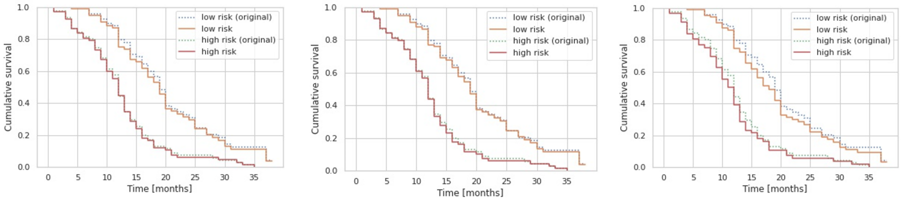

(A) (B) (C)

Figure S1: Kaplan-Meier survival curves for OS predictions of the Copenhagen dataset when segmentation errors are artificially introduced. The features included were the clinical and Hd95 features. The three figures show results for three different error levels: $max_{\%} = 5\%$ (A), 15% (B) and 25% (C). The original experiment from Fig. 6 (A) is shown with dotted lines.

2 Consistency of feature selection across cross-validation folds

In the interest of conciseness, selection of the proposed Hd95 features was performed on the entire cohort in our experiments, i.e., outside of the cross-validation set-up. While this potentially introduces information leakage between the training and test data within each fold, here we show that the results are only minimally affected in practice. Specifically, we ran our experiments again, selecting the features *within* each fold this time, and recording the number of folds each feature was selected in. Fig. S2 shows the frequencies (proportion of the cross-validation folds) of selected features – also shown is a color indicating whether the features were selected on the entire dataset or not. As can be seen from these results, the feature selection is largely consistent across folds, and in alignment with the feature selection performed on the entire cohort.

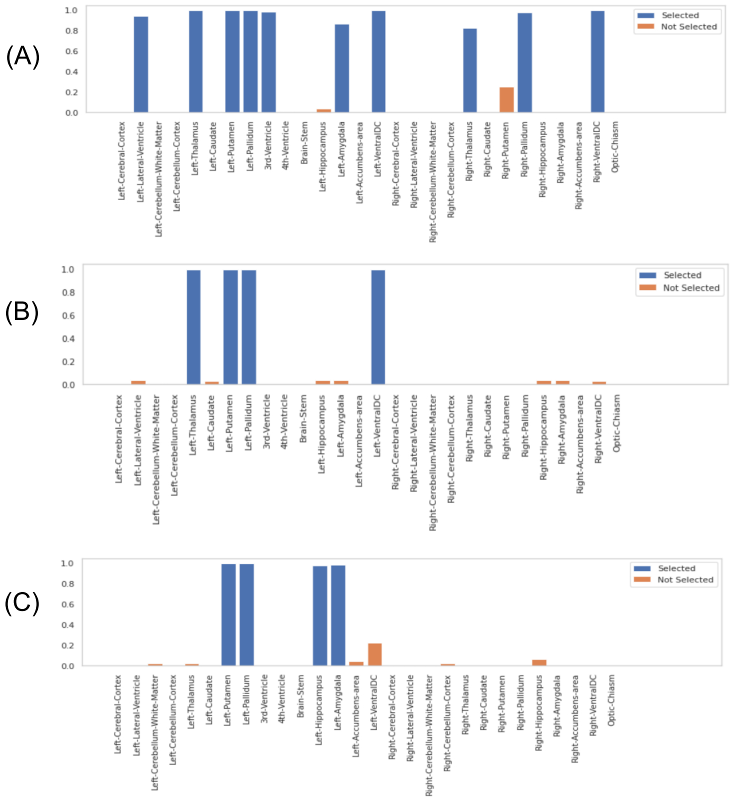

Figure S2: Frequency with which Hd95 features were selected across cross-validation folds on: (A) the Copenhagen data (OS), (B) the Copenhagen data (PFS), and (C) the BraTS20 data (OS). The colors indicate whether the features were also selected when a global feature selection was performed on the entire dataset instead.
